# JMJD6 in tumor-associated macrophage regulates macrophage polarization and cancer progression via STAT3/IL-10 axis

**DOI:** 10.1038/s41388-023-02781-9

**Published:** 2023-08-11

**Authors:** Siyuan Chen, Manni Wang, Tianqi Lu, Yu Liu, Weiqi Hong, Xuemei He, Yuan Cheng, Jian Liu, Yuquan Wei, Xiawei Wei

**Affiliations:** Laboratory of Aging Research and Cancer Drug Target, State Key Laboratory of Biotherapy and Cancer Center, National Clinical Research Center for Geriatrics, West China Hospital, Sichuan University, No.17, Block3, Southern Renmin Road, Chengdu, Sichuan 610041 People’s Republic of China

**Keywords:** Cancer microenvironment, Tumour immunology

## Abstract

The tumor-associated macrophage (TAM) is the most abundant group of immune cells in the tumor microenvironment (TME), which plays a critical role in the regulation of tumor progression and treatment resistance. Based on different polarization status, TAMs may also induce antitumor immune responses or immunosuppression. The present study identified JMJD6 (Jumonji domain-containing 6) as a novel modulator of TAM activation, the upregulation of which was associated with the immunosuppressive activities of TAMs. JMJD6 deficiency attenuated the growth of both Lewis lung carcinoma (LLC) tumors and B16F10 melanomas by reversing M2-like activation of macrophages, and sensitized tumors to immune checkpoint blockades (ICBs). Moreover, the JMJD6-induced inhibition of M2 polarization was potentially mediated by the STAT3/IL-10 signaling. These findings highlight the regulatory activities of JMJD6 in TAM polarization, and the therapeutic potential of JMJD6/STAT3/IL-10 axis blockades to enhance the efficacy of ICBs in cancer treatment.

## Introduction

Tumors are not simple stacks of malignant cells, but a complex system involving the tumor microenvironment (TME) including the extracellular matrix, vascular system and immune cells [1]. The tumor-associated macrophage (TAM) is the most abundant group of immune cells in the TME, which plays a critical role in the regulation of tumor progression and treatment resistance [2, 3]. TAMs are often characterized with the gene expression profile of M2 macrophages, such as the surface markers CD163, CD206 and Arg1, and the secretory factors IL-10 and TGF-β [4]. Substantial evidence suggests the protumoral effect of TAMs such as the promotion of tumor growth, angiogenesis, matrix remodeling, and tumor cell invasion and motility [5, 6]. TAM infiltration in some cases may induce immunosuppression by reshaping the immune microenvironment through the inhibition of the cytotoxic T lymphocyte (CTL) response [7]. It is thus intriguing to speculate whether the therapeutic targeting of TAMs could revert the suppressive TME by blocking the immunosuppressive activities of TAMs. On the other hand, despite numerous efforts, the molecular mechanism of TAMs polarization remains incompletely defined. The present study for the first time identified JMJD6 (Jumonji domain-containing 6) as a novel regulator of TAM activation which would serve as a therapeutic target and combination partner for ICBs in cancer.

The aberrant expression of JMJD6 has been reported in a wide panel of tumor types such as breast [8], hepatic [9], prostate cancer [10], colorectal [11] cancer and neuroblastoma [12]. The tumor expression of JMJD6 was also correlated with treatment resistance [13]. For instance, JMJD6 has been identified as a predicting marker for tumor response to tamoxifen endocrine therapy in breast cancer patients [14]. Moreover, with its arginine demethylation activities, JMJD6 is involved in multi-organ autoimmunity, modulating immune pathways such as Toll-like receptor related signal transduction. JMJD6 regulates the level of medullary thymic epithelial cell (mTEC) mature proteins via its ability to induce intron retention and the release of the Aire gene, which is required for self-immunity in the thymus [15].

JMJD6 was initially conceived as a phosphatidylserine receptor (PSR, Ptdsr) expressed on the surface of macrophages, mediating the phagocytosis of apoptotic cells [16]. In recent years, broader localization of JMJD6 was detected including the nucleus [17], cytoplasm, and extracellular matrix [18], and its target proteins including histones [19], non-histones p53 [20] and protein associated with RNA-splicing [21]. A recent report provided new insights to the mechanisms of JMJD6-mediated breast cancer progression. The genetic inhibition of JMJD6 decreased ANXA1 expression, preventing M2 polarization of macrophage and tumor aggressiveness [22]. This study aims to identify the role of JMJD6 in macrophage polarization and the remodeling of tumor microenvironment composition. We detected an upregulated JMJD6 level in TAMs which led to an immunosuppressive phenotype of TAMs. JMJD6 deficiency could attenuate the growth of both LLC tumors and B16F10 melanomas by reversing M2-like activation of macrophages, potentially mediated by STAT3/IL-10 signaling. Moreover, therapeutic inhibition of JMJD6 sensitized tumors to ICB treatment. Thus, the JMJD6/STAT3/IL-10 axis is a key switch in TAMs activation and a promising combination partner for ICBs.

## Results

### JMJD6 is upregulated in TAMs and indicates poor prognosis of cancer

**Fig. 1:**
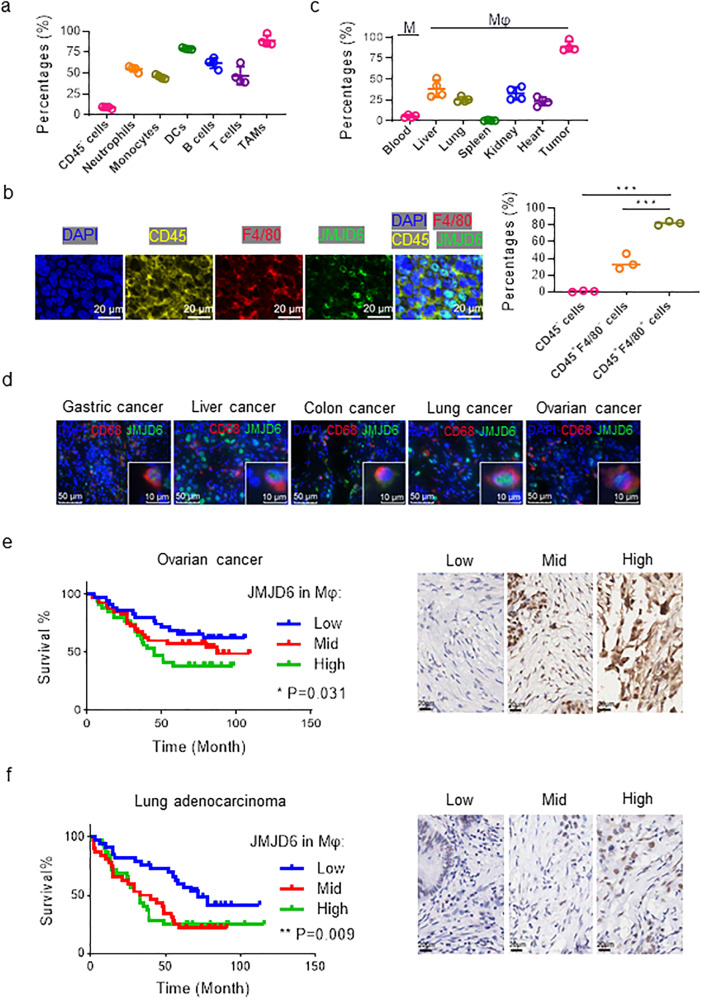
JMJD6 is upregulated in TAMs, and correlates with the cancer prognosis. **a** JMJD6 expression in tissue macrophages (monocytes in blood) was detected by flow cytometry, *n* = 4. **b** Representative immunofluorescence images of tumor sections from mice subcutaneously implanted with LLC cells, labeled by CD45 (yellow), F4/80 (red), JMJD6 (green) and DAPI (blue). Scale bars are marked in individual images. **c** JMJD6 expression in tumor cells (CD45^-^), tumor-infiltrating neutrophils (CD45^+^ CD11b^+^ Ly6G^−^ Ly6C^+^), monocytes (CD45^+^ CD11b^+^ Ly6G^−^ Ly6C^+^), DCs (CD45^+^ CD11b^+^ F4/80^−^ CD11c^+^ MHC-II^+^), T cells (CD45^+^ CD3^+^), B cells (CD45^+^ CD19^+^), and TAMs (CD45^+^ CD11b^+^ F4/80^+^) was detected by flow cytometry, *n* = 4. **d** Representative immunofluorescence images of tumor tissues from clinical samples. As marked in images, sections were labeled with CD68 (red), JMJD6 (green), DAPI (blue). Scale bars are marked in individual images. Survival curves of ovarian cancer patients (**e**) and lung adenocarcinoma patients (**f**) grouped by JMJD6 expression in stromal cells (left panel), analyzed by Logrank test for trend. Immunohistochemistry images were representative regions of JMJD6 expression in the stromal cells (right panel). Data are represented as mean ± SD. *P < 0.05, **P < 0.01.

The TME is characterized by the infiltration of a series of immune cells. To understand the role of JMJD6 in cancer, the expression levels of JMJD6 in different tumor-infiltrating cell subgroups were evaluated with flow cytometry assays in Lewis lung carcinoma (LLC) tissues (Fig. 1a). Among the seven types of tumor-infiltrating cells including tumor cells (CD45^-^), neutrophils (CD45^+^ CD11b^+^ F4/80^-^ Ly6C^mid/+^), monocytes (CD45^+^ CD11b^+^ Ly6G^−^ Ly6C^+^), DCs (CD45^+^ CD11b^+^ F4/80^−^ CD11c^+^ MHC-II^+^), T cells (CD45^+^ CD3^+^), B cells (CD45^+^ CD19^+^), and TAMs (CD45^+^ CD11b^+^ F4/80^+^), TAMs displayed the highest JMJD6 expression level (Fig. 1a, Supplementary Fig. S1a, b). Notably, although tumor cells constitute the majority of tumor bulks, the average JMJD6 expression level in tumor cells was significantly lower compared with tumor-infiltrating immune cells (Fig. 1a). The immunofluorescence (IF) staining results verified the presence of JMJD6 in TAMs (CD45^+^ F4/80^+^) (Fig. 1b). Compared with tumor cells (CD45^−^) and other immune cells (CD45^+^ F4/80^−^), TAMs (CD45^+^ F4/80^+^) exhibited higher JMJD6 expression. Additionally, JMJD6 expression of TAMs, monocytes in blood or macrophages in vital organs was measured by flow cytometry, which suggested that TAMs had higher JMJD6 expression than macrophages in other tissues or monocytes in blood (Fig. 1c, Supplementary Fig. S1c).

To validate the results in cancer patients, we assessed the expression levels of JMJD6 with immunofluorescence in tumor stroma of paired human tumors and adjacent normal tissues. Consistent results were observed that JMJD6 was widely expressed in tumor stromal cells of multiple cancer types including gastric, liver, colon, lung and ovarian cancer (Fig. 1d). To identify the prognostic value of JMJD6 expression in tumor stromal cells, tumor tissues from 109 ovarian cancer patients and 97 lung cancer patients were collected and classified according to their JMJD6 expression in stromal cells as low, medium, and high expression groups. Patients with higher JMJD6 expression in stromal cells had significantly worse survival compared with those with low or medium expression (Fig. 1e, f). These results suggested that JMJD6 was upregulated in tumor stromal cells that are mainly consisted of macrophages [23], and was correlated with the worse prognosis of cancer patients.

### JMJD6 is upregulated in macrophages stimulated by tumor-conditioned medium and promotes macrophage activation

**Fig. 2:**
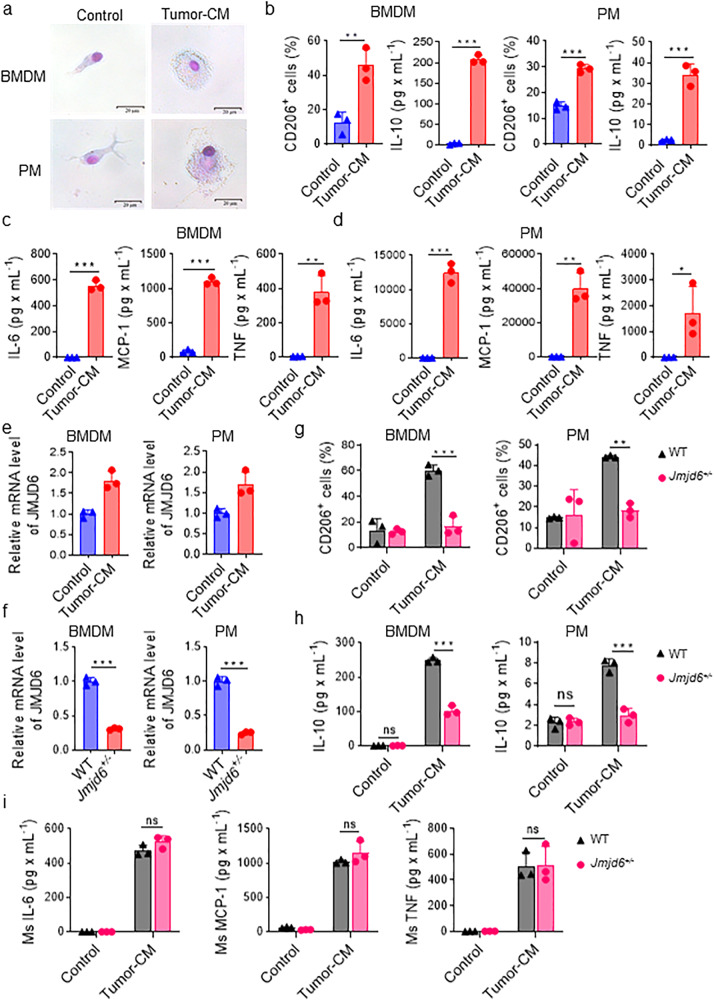
JMJD6 is upregulated in macrophages stimulated by tumor-conditioned medium and promotes macrophage activation. **a** Representative images after Giemsa staining of BMDM and PM treated by normal medium or tumor-CM. **b** CD206 and IL-10 expression on BMDMs and PMs treated by normal medium or tumor-CM were analyzed by flow cytometry and cytometric beads array (CBA), respectively. CBA was conducted to measure expression level of IL-6, MCP-1, IL-10 and TNF in the supernatant of the TC-Mφ from BMDMs (**c**) and PMs (**d**). **e** mRNA expression of JMJD6 was detected by qPCR in PMs and BMDMs stimulated by tumor-CM or not. **f** mRNA expression of JMJD6 was detected in TC-Mφ from BMDMs and PMs of WT or *Jmjd6*^*+/−*^ mice. **g** Flow analysis reveals the expression of M2 biomarkers CD206 in BMDMs and PMs stimulated by tumor-CM or not. **h** The secretion level of IL-10 in BMDMs and PMs stimulated by tumor-CM or not was detected by CBA assays. **i** BMDMs from WT or *Jmjd6*^*+/−*^ mice were cultured in Tumor-CM for 48 h, and the secretion level of IL-6, MCP-1, and TNF was detected by CBA. Data represent mean ± SD (*n* = 3). ns: no significant difference, *P < 0.05; **P < 0.01; ***P < 0.001.

In order to mimic the effect of tumor cells on macrophages in vivo, we utilized tumor-conditioned medium (tumor-CM) containing supernatants from tumor cell culture to stimulate mouse primary macrophages (M0), including primary bone marrow-derived macrophages (BMDM) and peritoneal macrophages (PM) (Supplementary Fig. 2a). Compared with macrophages cultured in normal medium, tumor-conditioned macrophages (TC-Mφ) stimulated by tumor-CM were transformed from an elongated, branched appearance to a rounder shape with vacuoles in the cytoplasm (Fig. 2a). The expression of TAM-associated markers CD206 and the level of IL-10 in TC-Mφ was next evaluated with flow cytometry and CBA analysis, respectively. Results demonstrated that CD206 was upregulated in TC-Mφ and that TC-Mφ produced expressed higher levels of IL-10 than M0 (Fig. 2b). Pro-inflammatory chemokines and cytokines (IL-6, MCP-1, and TNF) were increased in the medium supernatants of TC-Mφ (Fig. 2c, d). In addition, tumor-CM effectively upregulated JMJD6 expression in both BMDMs and PMs of mice (Fig. 2e).

The in vivo impact of JMJD6 expression on macrophage activation was analyzed using wide-type (WT) and *Jmjd6*^*+/−*^ mice. Following stimulation with tumor-CM, BMDMs and PMs of *Jmjd6*^*+/−*^ mice displayed significantly lower JMJD6 mRNA levels than WT mice (Fig. 2f). The expression of CD206 and secretion of IL-10 were both downregulated in macrophages and their medium supernatants collected from *Jmjd6*^*+/−*^ mice (Fig. 2g, h). These results indicated that the inhibition of JMJD6 in macrophages might prevent the tumor-CM-induced M2-like activation of these macrophages. Moreover, by detecting the secretion of M1-like chemokines and cytokines by macrophages from *Jmjd6*^*+/−*^ and WT mice, we found that *Jmjd6* deficiency failed to decrease the M1 activation of TAMs (Fig. 2i), suggesting that the inhibition of JMJD6 only affected the M2 activation, rather than M1 activation of TAMs. The production of IL-10 was significantly correlated with JMJD6 expression in macrophages as detected by western blotting (Supplementary Fig. S2b).

### Impaired tumor growth in *Jmjd6*^+/−^ mice is macrophage-dependent

**Fig. 3:**
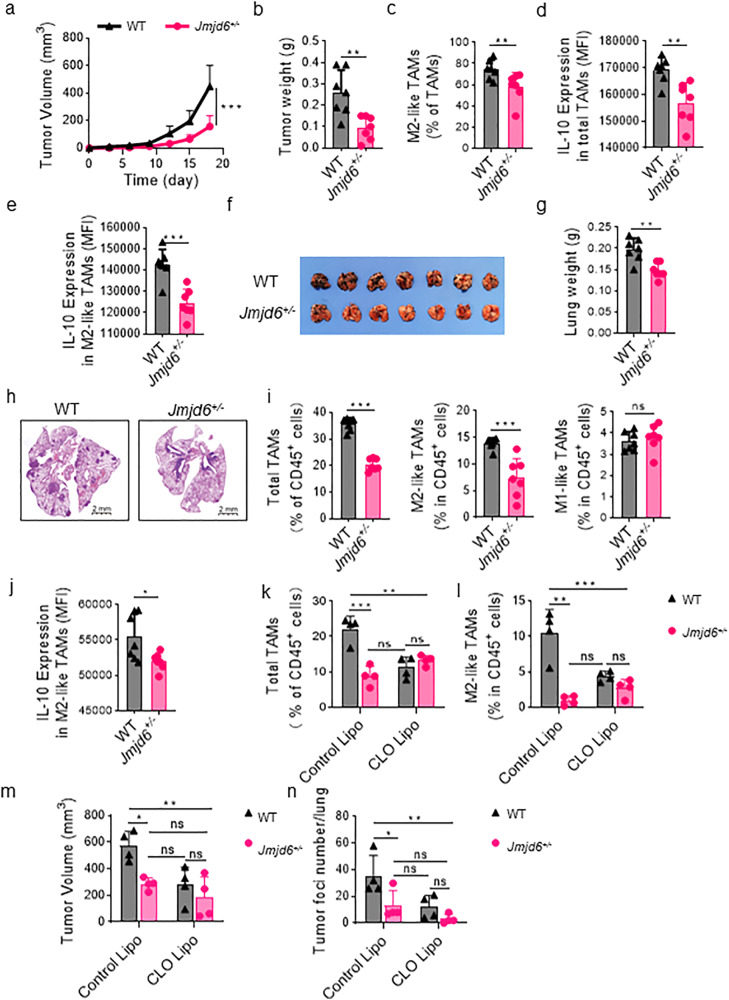
Impaired tumor growth in *Jmjd6*^+/−^ mice is macrophage-dependent. *Jmjd6*^*+/−*^ and WT mice were given subcutaneous infection of LLC lung cancer cells and tail vein injection of B16F10 melanoma cells. *n* = 7. **a** The tumor growth curve of subcutaneous LLC tumors from the two groups. **b** The weights of subcutaneous LLC tumors from the two groups. **c** The percentage of CD206^+^ M2-like TAMs in total TAMs infiltrating into the subcutaneous LLC tumors was analyzed by flow cytometry. IL-10 expression in total TAMs (**d**) and M2-like TAMs (**e**) infiltrating into the subcutaneous LLC tumors was analyzed by flow cytometry. **f** Mouse lungs at 21st day after B16F10 cell injection. **g** The weight of lungs. **h** Representative images H&E staining of lungs with B16F10 tumors. **i** The percentage of total TAMs, M2-like TAMs and M1-like TAMs in immune cells infiltrating into the lungs with B16F10 tumors was analyzed by flow cytometry. **j** IL-10 expression in M2 TAMs infiltrating into the lungs with B16F10 was analyzed by flow cytometry. **k**–**n** Mice were inoculated with tumor cells via tail vein 24 h after treatment of 200 μL clodronate liposomes (CLO Lipo). CLO Lipo was injected every four days to maintain the depletion of macrophages in vivo. Ctrl Lipo was used as control. *n* = 4. Flow cytometry results showed that CLO Lipo depleted TAMs (CD45^+^ CD11b^+^ F4/80^+^) (**k**) including M2-like TAMs (CD45^+^ CD11b^+^ F4/80^+^ CD206^+^) (**l**). **m** The effect of CLO Lipo treatment on the development of LLC subcutaneous tumors. Statistical diagram of tumor volume at the end of the experiment. **n** The effect of CLO Lipo treatment on B16F10 lung metastases. Statistical diagram of tumor foci numbers per lung at the end of the experiment. Data are shown as mean ± SD. ns: no statistical difference, *P < 0.05, **P < 0.01, ***P < 0.001.

To evaluate the role of JMJD6-expressing macrophages in tumor progression, we established two tumor models in WT and *Jmjd6*^*+/−*^ mice with subcutaneous injection of LLC lung cancer cells and intravenous injection of B16F10 melanoma cells. The tumor growth curves demonstrated a delayed growth of subcutaneous LLC tumors in *Jmjd6*^*+/−*^ mice compared with WT mice (Fig. 3a). At the end of the experiment, the average tumor weight of *Jmjd6*^*+/−*^ mice was significantly decreased (Fig. 3b). According to flow cytometry analyses, the proportion of CD206^+^ TAMs (M2-like TAMs) and IL-10 expression level of total TAMs and M2-like TAMs were decreased in *Jmjd6*^*+/−*^ mice compared with WT mice (Fig. 3c–e). Likewise, in the B16F10 pulmonary metastasis model, the *Jmjd6* knockdown impaired tumor growth as demonstrated by reduced number of lung metastasis and lung weights (Fig. 3f–h). Though the proportion of total TAMs in immune cells was decreased in *Jmjd6*^*+/−*^ mice, the decrease might be attributed to reduced M2-like TAMs rather than M1-like TAMs (Fig. 3i). Meanwhile, IL-10 expression in M2-like TAMs was suppressed in *Jmjd6*^*+/−*^ mice (Fig. 3j). Immunohistochemistry images of CD31 and Ki67 staining in subcutaneous LLC tumor sections were shown in Supplementary Fig 4. Tumors of *Jmjd6*^*+/−*^ mice displayed decreased CD31 and Ki67 positivity compared with WT mice, suggesting *Jmjd6* deficiency in macrophages resulted in decreased intratumoral vascular density and tumor cell proliferation.

To determine whether the antitumor efficacy of JMJD6 knockdown was macrophage-dependent, mice were administered with clodronate liposomes (CLO Lipo) to deplete macrophages during the course of tumor growth. As shown in Fig. 3k–l, CLO Lipo significantly depleted total TAMs (CD45^+^ CD11b^+^ F4/80^+^) and M2-like TAMs (CD45^+^ CD11b^+^ F4/80^+^ CD206^+^) in WT mice, whereas total TAMs and M2-like TAMs were not reduced in *Jmjd6*^*+/−*^ mice after intraperitoneal injection of CLO Lipo. The CLO Lipo treatment was also found to delay tumor growth and decrease the foci number of pulmonary metastasis in WT mice, which could not be observed in *Jmjd6*^*+/−*^ mice (Fig. 3n, m). Interestingly, we found that CLO Lipo reduced the difference in tumor growth between WT mice and *Jmjd6*^*+/−*^ mice (Fig. 3n, m). These results indicated that the impaired tumor growth in *Jmjd6*^*+/−*^ mice was dependent on macrophages.

### Tumor-conditioned medium upregulates JMJD6 expression in human monocyte cell line

**Fig. 4:**
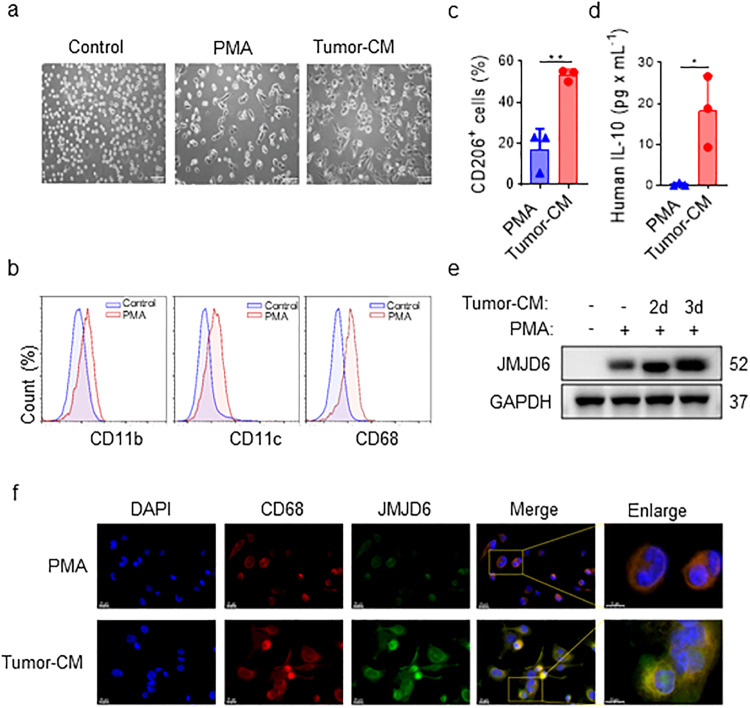
Tumor-CM activates THP-1 cells and upregulates JMJD6 expression. **a** Morphology of THP-1 cells cultured in normal medium, with or without PMA and tumor-CM (H460) for 48 h. Scale bar, 100 μm. **b** The changes of macrophage related markers on the surface of THP-1 cells were detected by flow cytometry after PMA induction. Representative results. **c** CD206 expression of TAM surfaces were detected by flow cytometry after tumor-CM induction. **d** The secretion of IL-10 in the TAM culture supernatant detected by CBA assays. **e** THP-1 cells were treated with PMA alone or tumor-CM for 2 or 3 days, followed by JMJD6 protein level detection. **f** Representative images of immunofluorescence staining of CD68 (red), JMJD6 (green) and DAPI (blue) in THP-1 cells treated with PMA alone or tumor-CM for 2 days, the scale bars 10 μm and 20 μm (right panel) as indicated in figures. Data are shown as mean ± SD (*n* = 3). *P < 0.05, **P < 0.01.

An acute monocytic leukemia cell line, THP-1 was used to investigate the role of JMJD6 in human monocytes. THP-1 cells activated by either PMA alone or together with tumor-CM from human tumor cells, were named M0-like THP-1 and TAM-like THP-1, respectively. Figure 4a presented the morphological changes of naïve THP-1, M0-like THP-1 and TAM-like THP-1. In contrast to the loosely suspended round THP-1 cells, M0-like THP-1 macrophages differentiated into a tightly adherent state, and TAM-like THP-1 macrophages displayed a fully differentiated morphology with an elongated, pseudopodia-like shape (Fig. 4a). Additionally, flow cytometry results confirmed the increased expression of macrophage differentiation markers including CD11c, CD11b and CD68 in PMA-induced M0-like THP-1 macrophages (Fig. 4b). Moreover, we also observed an increase in expression of CD206, a marker of TAMs, and the production of IL-10 in tumor-CM-induced TAM-like THP-1 compared with M0-like THP-1 macrophages (Fig. 4c, d, Supplementary Fig. 6a, b). Notably, western blot results showed that tumor-CM effectively elevated JMJD6 expression level, which was also found to be increased in TC-Mφ cells compared with M0-like THP-1 cells (Fig. 4e, Supplementary Fig. 6c). Immunofluorescence staining further confirmed the upregulated JMJD6 level in TC-Mφ cells (Fig. 4f).

### JMJD6 knockdown inhibits the activation of human monocytes

**Fig. 5:**
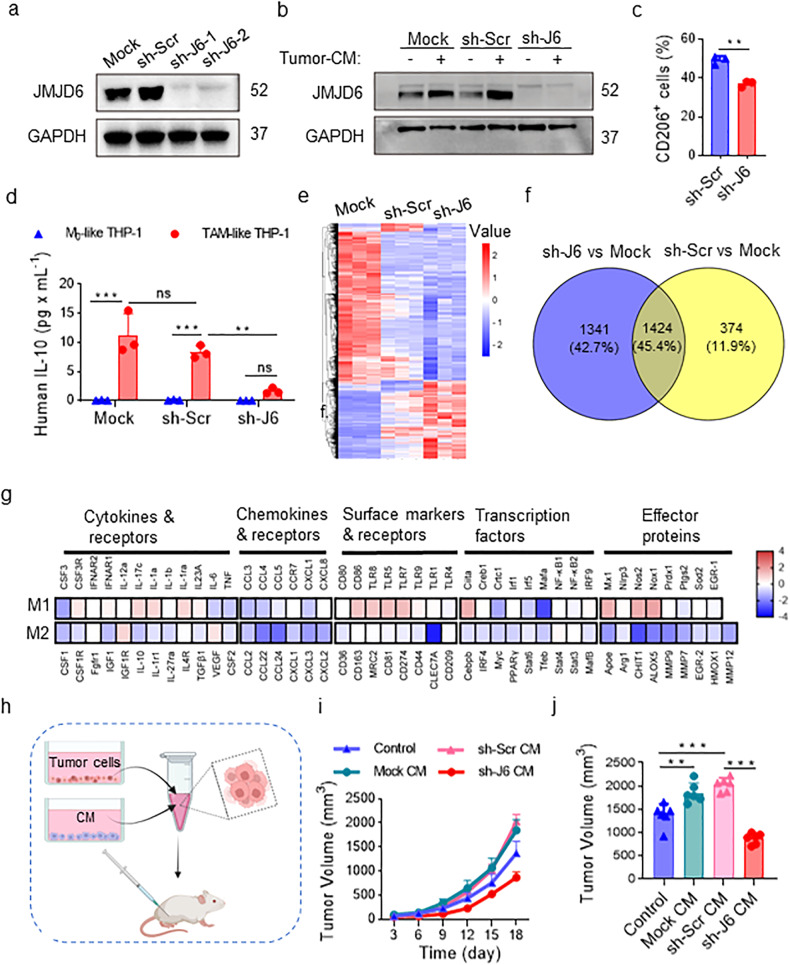
shRNA-induced JMJD6 inhibition in THP-1 cells alters macrophages phenotypes. **a** JMJD6 protein level was detected in THP-1 cells after shRNA interference. **b** Tumor-CM stimulation induced the increase of JMJD6 expression in the control groups, but did not change the low expression of JMJD6 in sh-J6 THP-1 cells. **c** CD206 on the TC-Mφ surfaces was analyzed by flow cytometry, *n* = 3. **d** IL-10 secretion in the culture medium of TC-Mφ was detected by CBA, *n* = 3. **e** RNA-seq heatmap of differentially expressed genes among Mock, sh-Scr and sh-J6 TAM-like THP-1 cells. 3139 differentially expressed genes were clustered by sample (column) and gene (row). **f** Venn diagram represented DEGs from two comparisons of sh-J6 vs Mock and sh-Scr vs Mock. **g** Heatmap of 87 genes related to M1 or M2 activation grouped by functional characteristics. Data were normalized to sh-Scr. **h**–**j** NOD-SCID mice were inoculated subcutaneously with 5 × 10^6^ human lung cancer H460 cells resuspended in conditioned medium (CM) of Mock, sh-Scr or sh-J6 TAM-like THP-1 cells, RPMI-1640 medium without additive agents (Control). The experiment was terminated on the 21st day. *n* = 6. **h** Schematic diagram. **i** Tumor growth curve. **j** Statistical diagram of tumor volumes at the end of the experiment. Data represent mean ± SD. ns: no statistical difference, *P < 0.05, **P < 0.01, ***P < 0.001.

To further asses the role of JMJD6 in THP-1 activation, we interfered JMJD6 expression in THP-1 cells with JMJD6 mRNA-targeting short hairpin RNA (shRNA-JMJD6, sh-J6). The Mock (blank) group and sh-Scrambled group (transduced with lentivirus expressing scrambled shRNA) were used as control groups throughout all shRNA-involved experiments. The qPCR results confirmed the decrease of JMJD6 expression in mRNA level caused by sh-J6 treatment (Supplementary Fig. 7a). Western blot assays indicated that sh-J6 induced a significant reduction in JMJD6 protein level whereas sh-Scr treatment had no effect on JMJD6 expression (Fig. 5a). To examine the role of JMJD6 in THP-1 activation, we detected JMJD6 expression in sh-Scr- or sh-J6- transfected THP-1 cells following tumor-CM stimulation (Fig. 5b). The sh-J6 group showed low JMJD6 expression even with tumor-CM stimulation, and the control groups both showed higher JMJD6 expression following tumor-CM stimulation. Consistent with the findings on mouse macrophages, the CD206 positivity of sh-J6 THP-1 cells was lower than that of sh-Scr group (Fig. 5c). Furthermore, IL-10 secretion was significantly decreased in sh-J6 TAM-like THP-1 cells compared with TAM-like THP-1 cells in control groups (Fig. 5d), whereas no difference was observed in the secretion of M1-like chemokines and cytokines between sh-J6 TAM-like THP-1 cells and control cells (Supplementary Fig. 7b).

To identify differentially expressed protein-coding genes in JMJD6-knockdown TAM-like THP-1 cells, RNA sequencing (RNA-Seq) was performed in TAM-like THP-1 cells transfected with blank, sh-Scr and sh-J6. The heatmap demonstrating the expression pattern of differentially expressed genes (DEGs) was presented in Fig. 5e. A total number of 2765 and 1798 DEGs were extracted in sh-J6 vs Mock group, and in sh-Scr vs Mock group, respectively (Supplementary Fig. 7c). The venn diagram summarized the 1424 DEGs overlap between the two comparisons (Fig. 5f). Based on the RNA-Seq results, we identified a global upregulation of M1 related genes and a global downregulation of M2 related genes in JMJD6-knockdown group (Fig. 5g).

To identify the role of JMJD6 expression in the antitumor effect of TAM-like THP-1 cells, we collected conditioned medium (CM) of Mock, sh-Scr or sh-J6 TAM-like THP-1 cells, and resuspended human lung cancer H460 cells with the CM, which was then inoculated subcutaneously in NOD-SCID mice (Fig. 5h). Compared with tumors formed by non-treated tumor cells, the volume of tumors formed by H460 cells incubated with supernatant of Mock and sh-Scr group were significantly increased. However, the tumor-promoting effect of sh-Scr group was reduced by sh-J6 TAM-like THP-1 supernatants (Fig. 5i–j). These results confirmed the tumor-promoting effect of TC-Mφ, which can be reversed by JMJD6 silencing, suggesting the regulatory role of JMJD6 in the protumoral activities of TC-Mφ.

### JMJD6 upregulates IL-10 expression of TAM via STAT3 pathways

**Fig. 6:**
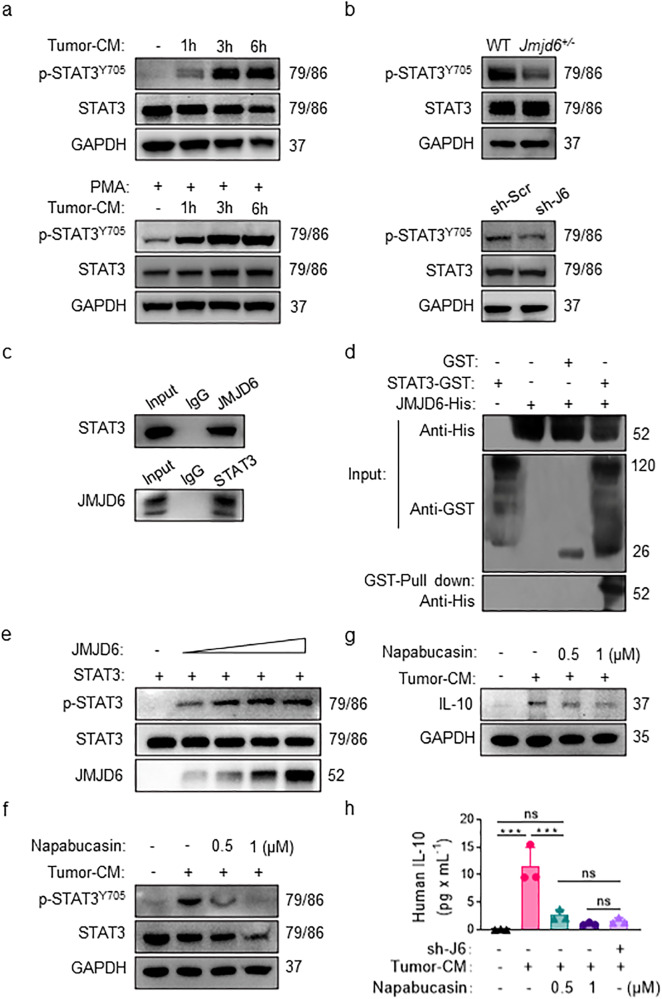
JMJD6 regulates macrophage activation through STAT3/IL-10 pathway. **a** Western blot assays on the level of phosphorylated STAT3 in total protein extracts from BMDMs (the up panel) or M0-like THP-1 cells (the down panel). **b** The expression levels of STAT3 and p-STAT3^Y705^ were detected in total protein extracted from BMDMs (the up panel) or M0-like THP-1 cells (the down panel) after 6 h stimulation by tumor-CM. **c** Co-IP assay on the expression of STAT3 protein and JMJD6 protein in TAM-like THP-1 cells. **d** GST pull-down assays on JMJD6-His and STAT3-GST. **e** Western blot assays on the purified recombinant STAT3 proteins (1–7.5 μg/lane) were incubated with purified recombinant JMJD6 proteins (1–7.5 μg/lane) in an in vitro phosphorylation system. **f** Inhibitory effect of napabucasin on phosphorylation of Y705 residue of STAT3 protein in macrophages. **g** Napabucasin inhibited the production of IL-10 protein in tumor-CM-stimulated macrophages. **h** Napabucasin inhibited the secretion of IL-10 in macrophage supernatant. Data represent mean ± SD (*n* = 3). ns: no significant difference, ***P < 0.001.

To investigate the underlying mechanisms of JMJD6-modulated macrophage activation, we analyzed the expression level of JMJD6 and M2-polarizing cytokines IL-10 in tumor-CM-activated macrophages. The identified positive correlation between JMJD6 and IL-10 expression leads to the hypothesis that IL-10 modulation by JMJD6 might be mediated by the STAT3 pathway. Western blot assay suggested the accumulation of p-STAT3^Y705^ in tumor-CM-activated macrophages in a time-dependent manner, with the most evident accumulation observed in macrophages following 6 h of stimulation (Fig. 6a). The phosphorylation level of STAT3^Y705^ was decreased in TAMs derived from BMDM of *Jmjd6*^*+/−*^ mice compared with those from WT mice, and STAT3^Y705^ expression was also downregulated in sh-J6 THP-1 macrophages compared with sh-Scr THP-1 (Fig. 6b). To determine the action mechanism of JMJD6 on STAT3, the co-immunoprecipitation (Co-IP) assay was performed which validated the interaction between JMJD6 and STAT3 (Fig. 6c). GST pull-down experiments demonstrated the direct binding between JMJD6 and STAT3 (Fig. 6d). We then performed the tyrosine kinase assay by incubating recombinant STAT3 with different doses of recombinant JMJD6 and assessing the phosphorylation of STAT3. The tyrosine kinase assay suggested that JMJD6 induced STAT3 phosphorylation in a dose-dependent manner in the in vitro system (Fig. 6e).

To elucidate whether the increased induction of IL-10 by tumor-CM in THP-1 cells was p-STAT3^Y705^ dependent, napabucasin was used as a selectively inhibitor of STAT3 synthesis, phosphorylation and activation. The expression levels of STAT3 and p-STAT3^Y705^ in TAM-like THP-1 cells were analyzed after treating THP-1 cells with napabucasin for 24 h. Napabucasin significantly reduced the expression of p-STAT3^Y705^ in a dose-dependent manner without affecting the total STAT3 protein levels in TAM-like THP-1 cells (Fig. 6f), and a dose-dependent decrease in IL-10 by napabucasin with the most significant decrease observed in TAM-like THP-1 cells treated with 1 μM napabucasin (Fig. 6g). The CBA assay was performed on the supernatants from THP-1 cell culture, and revealed a lower level of IL-10 production by TAM-like THP-1 cells treated with napabucasin (Fig. 6h).

### JMJD6 knockdown enhances PD-1-sensitivity in mice

**Fig. 7:**
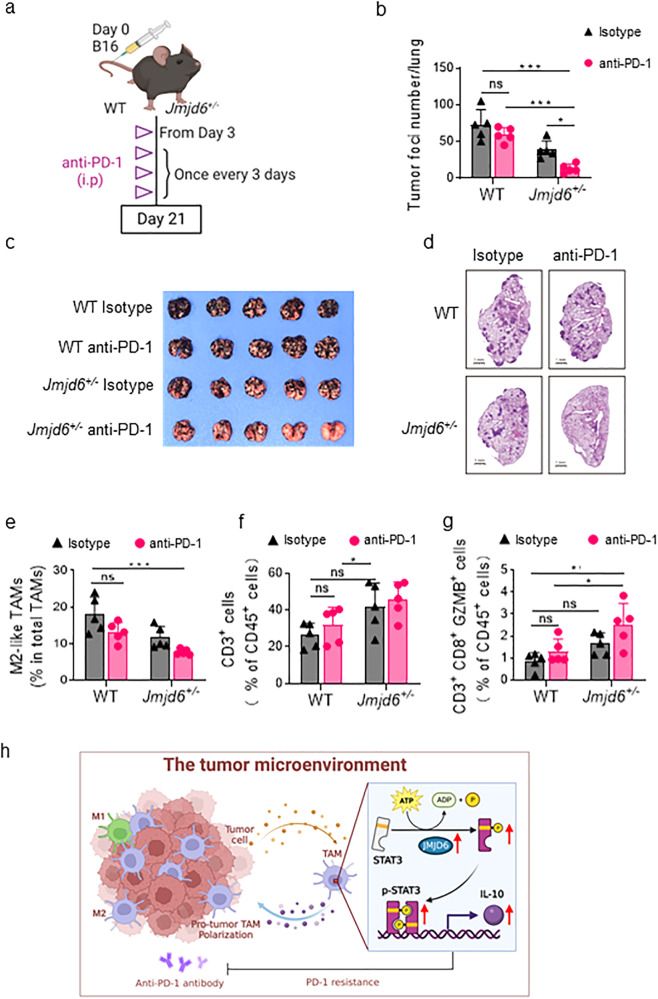
JMJD6 knockdown enhances PD-1-sensitivity in mice. **a** Schematic diagram of anti-PD-1 treatment on WT and *Jmjd6*^*+/−*^ mice B16F10 lung metastasis tumor model. **b** Lung photo at the end of the experiment. **c** Statistical results of the number of lung tumor nodules in mice. **d** Representative images of mouse lungs H&E staining. **e** The infiltration of M2-like TAMs (CD206^+^ MHC-II^-^), detected by flow cytometry. **f** The infiltration of CD3^+^ total T cells, detected by flow cytometry. **g** The infiltration of CD8^+^ GZMB^+^ T cells, detected by flow cytometry. **h** Illustration of mechanism for JMJD6/STAT3/IL-10 axis. Data represent mean ± SD (*n* = 5). ns: no significant difference, *P < 0.05, **P < 0.01, ***P < 0.001.

Based on the regulatory role of JMJD6 in immunosuppression, we investigated whether targeting JMJD6 in macrophages had a synergistic effect with PD-1 blockade in inhibiting tumor growth and metastasis. We treated melanoma-bearing WT and *Jmjd6*^*+/−*^ mice with in vivo anti-mouse PD-1 McAb once every 3 days from the third day of tumor inoculation (Fig. 7a). At the end of the experiment, *Jmjd6*^*+/−*^ mice treated with PD-1 blockade developed a smaller number of lung metastasis foci, whereas PD-1 blockade failed to induce significant tumor inhibition in WT mice (Fig. 7b, c). The HE staining further suggested that JMJD6 inhibition sensitized mice to PD-1 blockade in terms of tumor formation in lung (Fig. 7d). According to flow cytometry analyses, PD-1 McAb treatment reduced the number of M2-like TAMs in *Jmjd6*^*+/−*^ mice, but there was no significant change in the number of immunosuppressive M2-like macrophages in WT mice treated PD-1 McAb (Fig. 7e). In addition, an increase in the proportion of total T cells and activated cytotoxic T cells was observed in *Jmjd6*^*+/−*^ mice treated with PD-1 McAb (Fig. 7f, g). These results collectively suggested that *Jmjd6*^*+/−*^ mice were more sensitive to anti-PD-1 McAb, and the combination of JMJD6 inhibition and PD-1 blockade could induce a more potent antitumor immune response.

## Discussion

Immune cells and non-immune cells collectively compose the TME and determine the fate of tumors. By inducing stromal cell proliferation, migration, angiogenesis, and extracellular matrix deposition (ECM), the abundantly-infiltrated TAMs reshape the TME into one that favors tumor progression [24]. TAMs mainly present the M2 phenotype and are related to the poor prognosis of tumor patients [5, 25]. Thus, targeting M2-TAMs is a potential therapeutic strategy to reverse the immunosuppressive TME.

Current therapeutic strategies targeting TAMs mainly fall into four categories. (1) the inhibition the recruitment of TAMs to tumor sites by blocking various chemokines produced by tumor cells and stromal cells [26]; (2) the depletion of TAMs with CSF-1R antibodies [27]; (3) reprogramming TAMs by switching the M2 polarization state to M1 [23]; (4) targeting the immunoinhibitory molecules on TAMs such as leukocyte immunoglobulin-like receptor subfamily B (LILRB) [28] and PD-L1 [29]. Given the fundamental role of tissue-resident macrophages in maintaining homeostasis, the indiscriminative depletion of macrophages may cause serious damage to physiological functions [30–32]. By contrast, interfering the M2 polarization of TAMs is considered a safe option that effectively reduces the immunoinhibitory activities of TAMs and at the same time has no impact on the recruitment and maintenance of other macrophages [33]. The exact molecular mechanism underlying the switch of TAM polarization status remains incompletely defined. The present work investigated the role of JMJD6 in the polarization and antitumor function of TAMs, and discussed the potential therapeutic value of JMJD6-targeted strategies.

The aberrant expression of JMJD6 is associated with progression, prognosis and treatment resistance in multiple tumors such as breast [8], hepatic [9], prostate cancer [10] and neuroblastoma [12, 13]. In addition to the tumor expression of JMJD6, recent studies suggested that the correlation of JMJD6 with tumor prognosis was not only based on tumor cell-intrinsic mechanisms, but also the molding the TME. These results provided new insights to the mechanisms of JMJD6-mediated cancer progression that JMJD6 might promote the M2 polarization of macrophage and the subsequent tumor aggressiveness [22]. However, it remains incompletely defined whether and trough which mechanism JMJD6 regulates the activation of TAMs and the tumor microenvironment. To the best of our knowledge, this is the first research demonstrating the high expression of JMJD6 in tumor-infiltrating macrophages. To certify the regulatory role of JMJD6 in macrophage polarization, the macrophages used in this study were collected from different origins, including primary BMDMs, PMs, and human monocyte cell line THP-1. Given the known neonatal lethality of *Jmjd6*^*−/−*^ mice [34], the primary macrophages were isolated from *Jmjd6*^*+/−*^ mice.

It has been well-established that STAT3 signaling is involved in the regulation of immune response by macrophages [35], with a higher level of pro-inflammatory cytokines produced by macrophages of STAT3-knockout mice [36]. STAT3 is a key signaling molecule for macrophage polarization toward the M2 phenotype and is an important signal transducer for oncogenic proteins [37, 38]. In this study, we found that JMJD6 induced M2 macrophage polarization via the activation of the STAT3 signaling pathway. Accordingly, blocking the JMJD6-STAT3 axis by JMJD6-knockdown impaired tumor growth in vivo. The upregulated STAT3 in macrophages may also impair the antigen-specific T-cell response [39]. It is thus conceivable that targeting the JMJD6-STAT3 axis in TAMs could improve the antitumor immune reactions mediated by T cells. Though ICB treatments displayed promising efficacy in controlling tumor growth by unleashing the restricted T-cell–mediated activities against tumor cells [40], many patients ultimately develop resistance to ICB therapy, which may be attributed to the high infiltration of immune-suppressive myeloid cells such as TAMs [41, 42]. Whereas the single use of anti-PD-1 antibody failed to induce significant antitumor effect in this study, JMJD6 knockdown overcame the resistance of metastatic melanoma cells to anti-PD-1 treatment, suggesting the combination potential of JMJD6 inhibition with ICB therapies.

## Conclusions

Taken together, our study demonstrated that the M2 polarization of macrophages in TME is potentially mediated by the JMJD6/STAT3/IL-10 signaling (Fig. 7h). JMJD6 promotes the M2 activation and protumoral activities of macrophages, thereby establishing an immunosuppressive TME favoring tumor growth. Moreover, JMJD6 knockdown overcame the tumor resistance to anti-PD-1 treatment, making TAMs-targeted therapy a potential combination partner for ICB therapies.

## Materials and methods

### Cell lines and antibodies

THP-1 cells were cultured in complete Roswell Park Memorial Institute (RPMI) 1640 medium [supplemented with 10% FBS, penicillin (100 mg/mL) and streptomycin (100 mg/mL)], and 0.05 mM 2-mercaptoethanol (2-ME) was added to the medium to culture THP-1 cells. H460 cells and A549 cells were separately cultured in complete 1640 medium or complete Dulbecco’s modified agle’s medium (DMEM) [supplemented with 10% FBS, penicillin (100 mg/mL) and streptomycin (100 mg/mL)]. THP-1, A549 and H460 cells were recently authenticated by STR profiling. B16 and Lewis lung cancer (LLC, LL2) cells were cultured in complete DMEM medium. All the cells used in this study were tested for mycoplasma (Yeasen, 40612ES60). The antibodies we used: Anti-JMJD6: sc-28348 (human), sc-28349 (mouse); Anti-GAPDH: sc-32233, Santa Cruz, CA, USA; Anti-STAT3: 9139, CST; Anti-p-STAT3^Y705^: 145, CST; Anti-F4/80: ab16911, abcam; Anti-IL-10:ER1911-19; Anti-CD31: GB11063; Anti-Ki67: GB111499; Goat anti-Rabbit IgG (H + L) Secondary Antibody, HRP: Invitrogen, 31460; Goat anti-Mouse IgG (H + L) Secondary Antibody, HRP: Invitrogen, 31430.

### Animal experiments

All animal experiments were approved by Experimental Animal Ethics Committee of State Key Laboratory of Biotherapy, Sichuan University (NO.20190923034). The animal research included in the study were all conducted with randomization, allocation concealment, and blind outcome assessment. *Jmjd6*^*+/−*^ (B6.129S6-Del (11Jmjd6-Mettl23) 1Flv/J) mice were purchased from the Jackson Laboratory (Stock Number: 007666). C57BL/6 and NOD-SCID mice were purchased from Charles River (Beijing, China). In the animal experiments involving both *Jmjd6*^*+/−*^ and WT mice, the WT mice used as controls were the *Jmjd6*^*+/+*^ offspring of the same parent mice with *Jmjd6*^*+/−*^ mice, and were of the same age with *Jmjd6*^*+/−*^ mice, also with B6.129S6 background. All the experiments in vivo were performed on female mice aged 6–8 weeks. For subcutaneous tumor model, 1 × 10^5^ LLC cells suspended in 100 μL were injected at the right flank subcutaneously. Tumor was measured with electronic caliper (a: the smallest diameter, b: the largest diameter) every three days, and tumor volume was calculated as *a*^2^ × *b*/2. Mice were sacrificed on day 18 (18 days after implantation, n = 7 per group). For lung metastatic cancer model, 1 × 10^5^ B16 cells suspended in 100 μL were injected in the tail vein intravenously (i.v.). Mice were sacrificed on day 18 (18 days after implantation, *n* = 7 per group).

### In vivo macrophage depletion

Clophosome-A Liposomes (FormuMax Scientific, F70101C-A) were intravenously injected into mice once a week starting from day 5 after tumor inoculation. The control group was injected with the same volume of control liposomes at the same time.

### Clinical samples

Clinical samples and data of patients were obtained from Shanghai Outdo Biotech (National Engineering Centre for Biochip at Shanghai). The informed consent was obtained from patients and the study was approved by the Ethics Committee of National Human Genetic Resources Sharing Service Platform (permit number: 2005DKA21300). Tumor tissues and their adjacent normal tissue specimens were obtained from 109 ovarian cancer patients and 97 lung cancer patients. Tissues were stained using human JMJD6 antibody and the staining levels were scored by multiplying the intensity and the positive rate of staining, as described in the previous study [43].

### Peritoneal macrophages (PM) and bone marrow-derived macrophages (BMDM) isolation and culture

Euthanize C57BL/6 wild-type or *Jmjd6*^*+/−*^ mice, and retract their abdominal skin to expose the intact peritoneal wall. For the isolation of PM, 10 mL normal saline was injected intraperitoneally, and then fluid from peritoneum was aspirated with the same syringe. After centrifuging, the peritoneal cells were collected and cultured in complete RPMI-1640 medium for 2 h to allow the adhesion of macrophages. For the isolation of BMDM, the bone marrow was harvested from their femurs and tibia, and was filtered through a 70 μm cell strainer. Cells were cultured in complete RPMI-1640 medium with 20 ng/mL M-CSF for 5 days. For tumor-supernatant treatment, PMs and BMDMs were incubated in complete RPMI-1640 medium containing 20 ng/mL M-CSF and 10% B16 conditioned media for 2 days. For classical and alternative activation of macrophages, 100 ng/mL LPS (Sigma) + 20 ng/mL IFN-γ (Novoprotein) or 20 ng/mL IL-4 (Novoprotein) were added to the culture medium for 2 days, respectively.

### Giemsa staining

Giemsa Stain solution (Solarbio No: G4640) following the manufacturer’s protocol. Briefly, cells were cultured in 6-well plates and then fixed in cold methanol. Immerse the cells in the dye and placed at room temperature for 20 min. Giemsa staining was viewed under the optical microscope.

### TAM-Like THP-1 cells induction in vitro

THP-1 cells were induced to M0 macrophages (M0-like THP-1) by 150 ng/mL Phorbol-12-myristate-13-acetate (PMA, Sigma, P-8139) for 24 h, and then CD68, CD11c and CD11b expressions were detected by the flow cytometry. As for tumor-CM treatment, tumor-CM was prepared by mixing H460 (Fig. 4a–f) or A549 (Supplementary Fig. 6a–c) tumor cell culture supernatant (1/3 of the total volume) and 1640 complete medium. M0-like THP-1 was cultured for 6–48 h, according to the need of specific experiments.

### shRNA knockdown of JMJD6

JMJD6 knockdown was performed using lentiviral transduction. Briefly, THP-1 cells were grown in a 6-cm well to 70–80% confluence and then transfected with JMJD6 lentiviral shRNA constructs (GeneCopoeia). As for control, cells transfected with a scrambled shRNA (sh-Scr) was negative control, and the untreated cells were named Mock group. To generate robust and persistent JMJD6 knockdown, THP-1 cells were selected for puromycin resistance in media containing 4 μg/mL puromycin for 3 days and cultured with 2 μg/mL puromycin for the rest of the experiments.

### Immunohistochemistry and H&E staining

Immunohistochemistry analyses and H&E staining of tumor tissue section were performed as described previously [44]. Briefly, we dewaxed paraffin-embedded tissue sections by immersing tissues in graded concentrations of xylene and rehydrated them by graded concentrations of ethanol. Next, we blocked endogenous peroxide in dark and induced antigen-repairing. The nonspecific binding sites were blocked with goat serum, and then incubated t with specific primary antibody. The slides were then incubated with HRP-conjugated appropriate secondary antibody and streptavidin-biotin complex. We detected HRP with diaminobenzidine peroxide solution and counterstained cell nuclei with hematoxylin (BeyotimeInstitute of Biotechnology, Shanghai, China). For hematoxylin-eosin (H&E) staining, the tissue sections were hydrated and stained with HE Staining Kit (Solarbio).

### Immunofluorescence staining

Immunofluorescence of tumor tissue section was performed as described in the previous study [45]. For cyto-immunofluorescence staining, mouse primary cells or TAM-like THP-1 cells were seeded in six-well plates with pre-placed 24 mm glass slides (Shanghai WoHong Biotechnology, WHB-6-CS). The staining procedures were consistent with the tissue staining mentioned above.

### Quantitative real-time PCR (RT-PCR, qPCR)

Total RNA was isolated using Cell Total RNA Isolation Kit (FOREGENE, RE-03113), and Prime Script RT Kit (Takara, RR047A) was used for reverse transcription to cDNA. qPCR was performed by using SsoFast EvaGreen Supermix (Bio-Rad, USA, 1725201) by CFX Connect Real-Time PCR system (Bio-Rad, USA). The sequences of the primer sets were as follows:

**Table:** 

	Forward	Reverse
GAPDH	ACCCAGAAGACTGTGGATGG	ACATTGGGGGTAGGAACAC
JMJD6	GTTCCAGCTCGTCAGACTCG	TGCCCCTAAGACATGACCAC

### Western blot and co-immunoprecipitation assay

The cells were collected and washed with cold PBS buffer. For total cellular protein extraction, the cells were lysed in RIPA buffer (Beyotime), in which protease inhibitor cocktail (MedChemExpress, MCE) and phosphatase inhibitor cocktail (MCE) were added. The cells were grounded (Homogenizer KZ-II, Servicebio) and the supernatant was collected after centrifugation. The protein quantitative analysis of each cell lysate was performed with the Piece^TM^ Rapid Gold BCA Protein Assay Kit (Thermo Fisher, USA, 23225). Loading buffer (Beyotime) was then added to the cell lysate, and the mixture was denatured in a boiling bath. The same amounts of protein and molecular weight marker were loaded into the proper of sodium dodecyl sulfate‐polyacrylamide gel electrophoresis (SDS‐PAGE) gels of appropriate concentration, and transferred onto polyvinylidene difluoride (PVDF) membranes (Millipore). The membranes then were blocked with 5% milk. Next, the membranes were probed by specific antibodies overnight at 4 °C. And then, we washed the membranes, and incubated them with appropriate secondary antibodies. Band images were acquired by chemiluminescence (Bio‐Rad Laboratories). As for immunoprecipitation in brief, the incubation of cell lysates were performed first with appropriate specific antibodies for 12 h at 4 °C and then with protein-A/G–agarose beads for 2 h. After washing for five times with RIPA buffer, the precipitated complex was analyzed by western blot.

### Flow cytometry

For tumor microenvironment analyses, mouse tumors tissues and lungs containing tumor nodules were digested in 10 mL RPMI-1640 medium containing 10 mg collagenase (Gibco) and 1 U/mL DNase I (Keygentec, KGF008) at 37 °C with periodic vortex and inversion for 1 h. The suspensions further flew through 70 μm cell strainers and then the red blood cell lysis buffer was added to lyse red blood cells. Collected cells were stained with antibodies following per corresponding protocol. For intracellular staining, the surface antigens-labeled cells were fixed and permeabilized by Cytofix/Cytoperm Fixation/Permeabilization Kit (BD Pharmingen, 554715), and subsequent staining was performed following specific antibody protocols. Samples were acquired by using ACEA NovoCyte, and data was analyzed by NovoExpress software. For cultured cell lines and mouse primary macrophages, the samples were harvested by standing in trypsin (Gibco) and PBS at 37 °C for 3–5 min respectively, and stained with antibodies following procedures described above.

### GST pull-down assay

Protein STAT3-GST and protein JMJD6-His were subjected to GST pull-down test to verify whether protein STAT3-GST and protein JMJD6-His interact. GST protein was used as negative control. After reacting each sample in the input group with GST Resin, eluting with GSH, WB identification was performed to determine whether there was interaction between the validation protein STAT3-GST and the protein JMJD6-His.

### RNA sequencing

Tumor CM-primed THP-1 cells were treated with sh-Ctr, sh-J6 or blank, and then lysed with TRIzol reagent and stored at −80 °C, with three biological duplicates for each condition. The Agilent 2100 Bioanalyzer and RNA Nano 6000 Assay Kit (Agilent Technologies) was used to detect the integrity and concentration of RNA extracts. For the preparation of RNA-seq library, total RNA was purified by oligo (dT) beads and fragmented, followed by synthesis of first and second strand, 3′ ends adenylation and adapter ligation. The obtained samples were amplified by PCR subsequently to gel extraction. High throughput Illumina HiSeq 2500 (Illumina) sequencing was accordingly performed.

### In vitro tyrosine kinase assay

Purified recombinant STAT3 proteins were incubated with purified recombinant JMJD6 proteins for in vitro tyrosine kinase assay. JMJD6 was loaded at increasing amounts (1–7.5 μg/lane) and STAT3 was used at 7.5 μg/lane in the in vitro phosphorylation system containing 25 mmol/L Tris-HCl, pH 7.5, 20 μM ATP, EDTA-free protease inhibitor, phosphatase inhibitor cocktail, 2 mM DTT, 20 mM HEPES (pH 7.5), and 10 mM MgCl_2_. After for 30 min, the reaction was terminated by 5× loading buffer and heating for 10 min at 95 °C. These samples were analyzed in western blot analysis.

### Statistical analysis

All data were analyzed by GraphPad Prism 7.0. The significance of differences between two groups were determined by Student’s *t* test (parametric) and ANOVA multiple comparison tests. Statistically significant differences were labeled as: ns, no significance (*p* < 0.05 was considered as statistic significant), *P < 0.05, **P < 0.01, ***P < 0.001.

## Supplementary information

Supplementary figure legend

Supplementary figures

## Data Availability

The datasets in this study are available from the corresponding author on reasonable request.
