# Supplementary material for: JMJD6 in tumor-associated macrophage regulates macrophage polarization and cancer progression via STAT3/IL-10 axis
Source: Oncogene. 2023 Aug 11;42(37):2737–50. doi: 10.1038/s41388-023-02781-9 (PMC10491492; doi:10.1038/s41388-023-02781-9)
Supplement: Supplementary file 1 — Supplementary figure legend [file 41388_2023_2781_MOESM1_ESM.pdf]

Supplementary Figure 1. a, b. The method of gating immune cells in tumor.

c. The method of gating monocytes (M) from peripheral blood and various tissue macrophages.

Supplementary Figure 2. a. Schematic diagram of *in vivo* stimulation of primary bone marrow derived macrophages (BMDMs) and peritoneal macrophages (PMs). b. JMJD6 and IL-10 protein levels were detected in TC-Mφ from BMDMs.

Supplementary Figure 3. a. The method of gating TAMs infiltrated in subcutaneous LLC tumor tissues by flow cytometry. b: Representative images to show the percent of $CD206^+$ cells in TAMs infiltrated in subcutaneous LLC tumor tissues.

Supplementary Figure 4. a. Difference of angiogenesis in LLC subcutaneous tumor tissue between $Jmjd6^{+/-}$ mice and WT mice. The number of microvessels per area was calculated by CD31 immunohistochemical staining. The value of the average microvessel density (AMVD) manual counting under microscopy. Fifteen microscopic fields (each microscopic field was $0.1mm^2$) were selected at random for counting for each tumor sample. b. Difference in cell proliferation in LLC subcutaneous tumor tissue between $Jmjd6^{+/-}$ mice and WT mice. Representative images and statistic graph of the cell proliferation marker Ki67 immunohistochemistry detection. Scale bars are shown at the bottom left of individual images. Data represent mean ± SD (n=7). $***P < 0.001$.

Supplementary Figure 5. Analysis of macrophages in B16-F10 lung metastasis model by flow cytometry. a. The method of gating immune cells ($CD45^+$). b. TAMs ($CD11b^+$ $F4/80^+$) and its M1 ($MHC\text{-}II^+$) and M2 ($CD206^+$) phenotype. c. The method of gating

alveolar macrophages ($CD11c^{+}$ $CD11b^{-}$). d. The percentages of alveolar macrophages in $CD45^{+}$ immune cells. Data represent mean ± SD (n=7). ns: no significant difference.

Supplementary Figure 6.

a. CD206 expression of TAM surfaces were detected by flow cytometry after tumor-CM (A549) induction. b. The secretion of IL-10 in the TAM (inducted by A549 tumor-CM) culture supernatant detected by CBA assays. c. THP-1 cells were treated with PMA alone or tumor-CM (A549) for 2 or 3 days, followed by JMJD6 protein level detection.

Supplementary Figure 7.

a. JMJD6 mRNA expression in THP-1 cells interfered by shRNA was analyzed by qPCR. Data was relatively quantified to the untransfected shRNA group (Mock) as the standard. b. CBA detected the content of IL-6, VEGF and TNF in the culture medium. c. Statistics represented DEGs from two comparisons of sh-J6 vs Mock and sh-Scr vs Mock. Data represent mean ± SD (n=3). ns: no significant difference, $^{***}P < 0.001$.
