# Supplementary material for: JMJD6 in tumor-associated macrophage regulates macrophage polarization and cancer progression via STAT3/IL-10 axis
Source: Oncogene. 2023 Aug 11;42(37):2737–50. doi: 10.1038/s41388-023-02781-9 (PMC10491492; doi:10.1038/s41388-023-02781-9)
Supplement: Supplementary file 2 — Supplementary figures [file 41388_2023_2781_MOESM2_ESM.pdf]

Supplementary Figure 1.

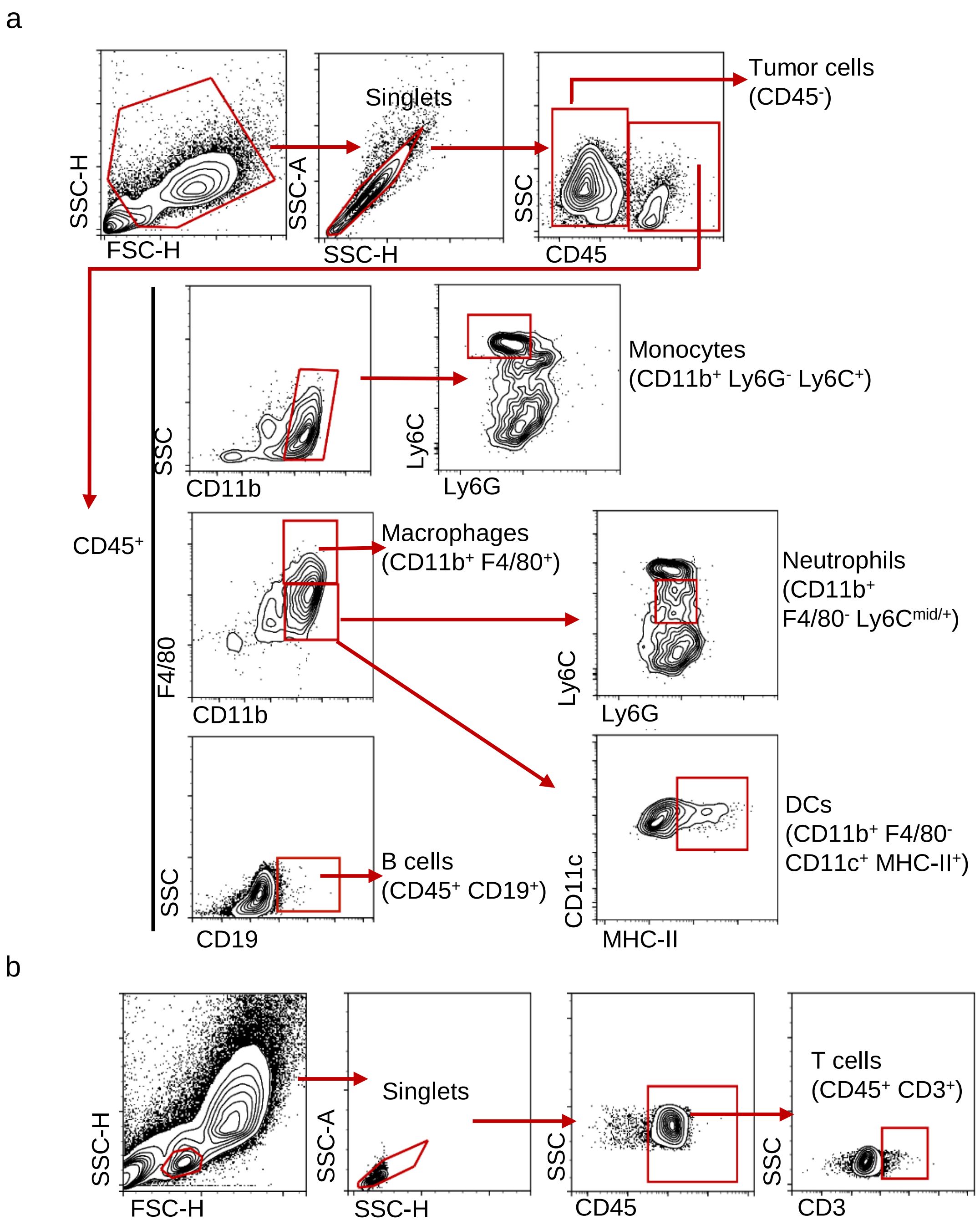

Supplementary Figure 1.

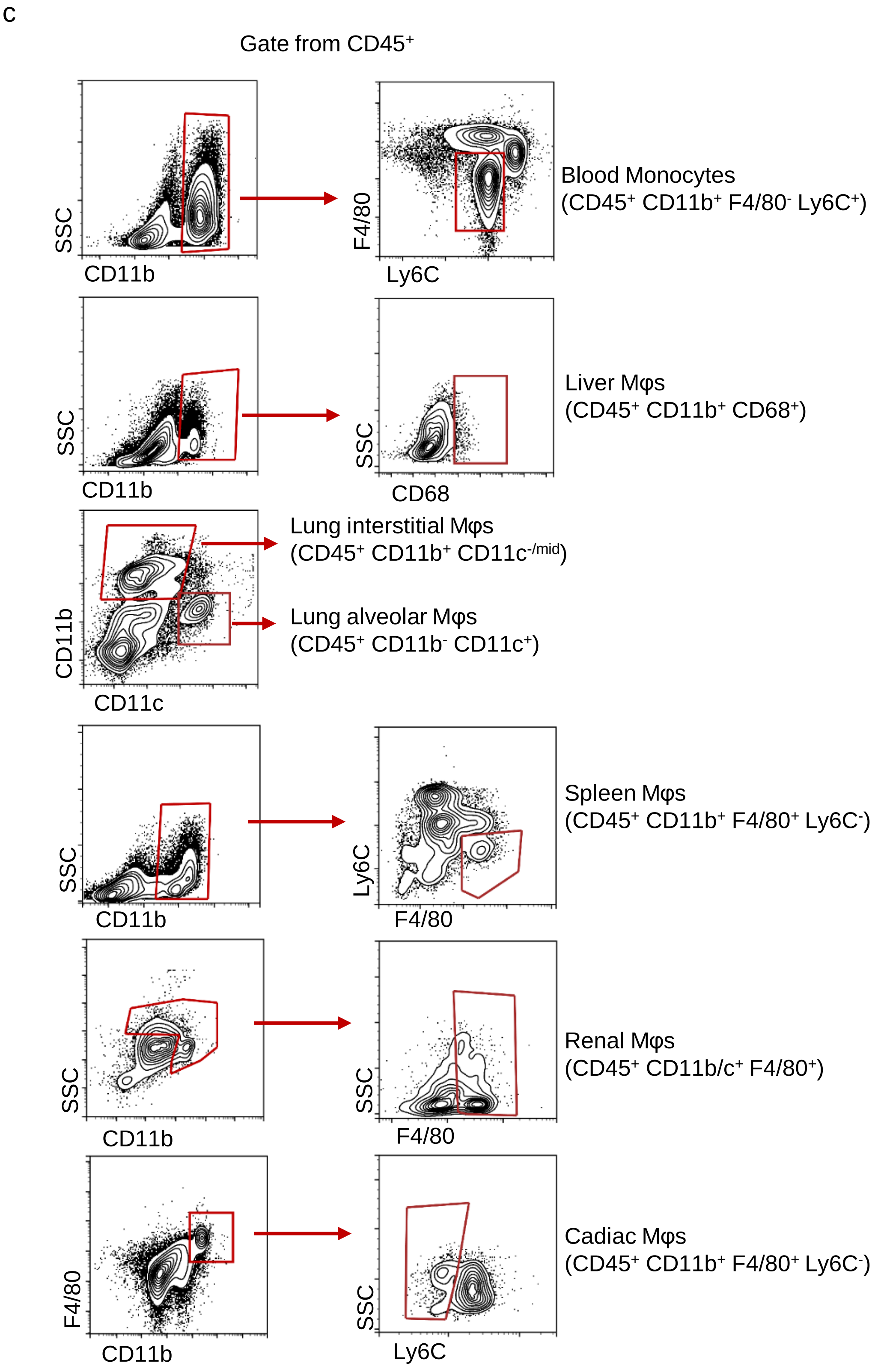

Supplementary Figure 2.

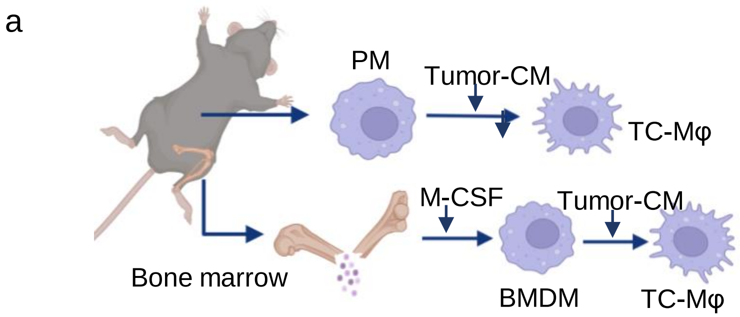

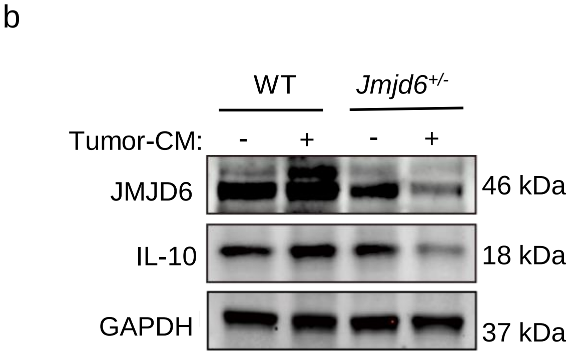

Supplementary Figure 3.

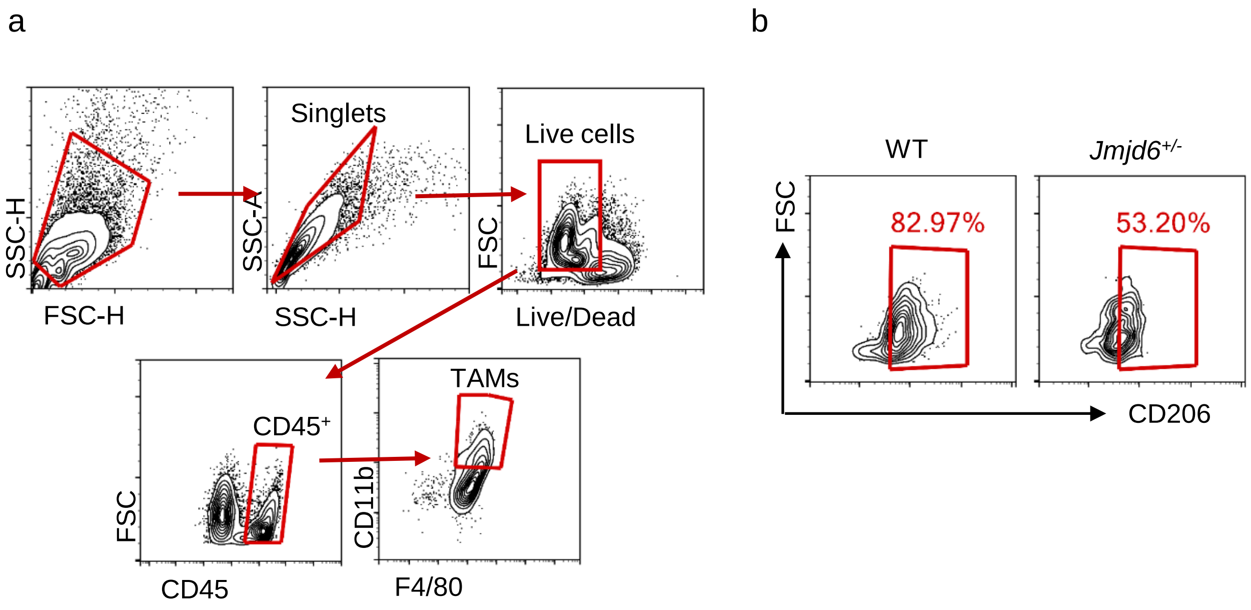

Supplementary Figure 4.

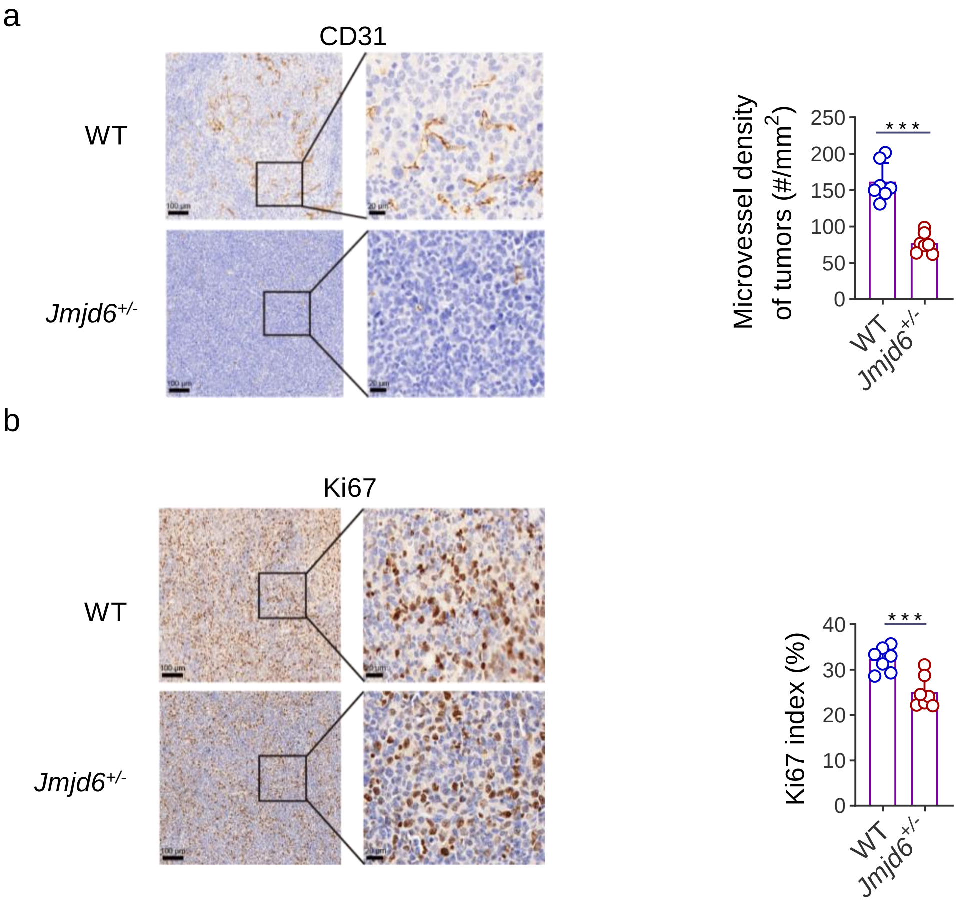

Supplementary Figure 5.

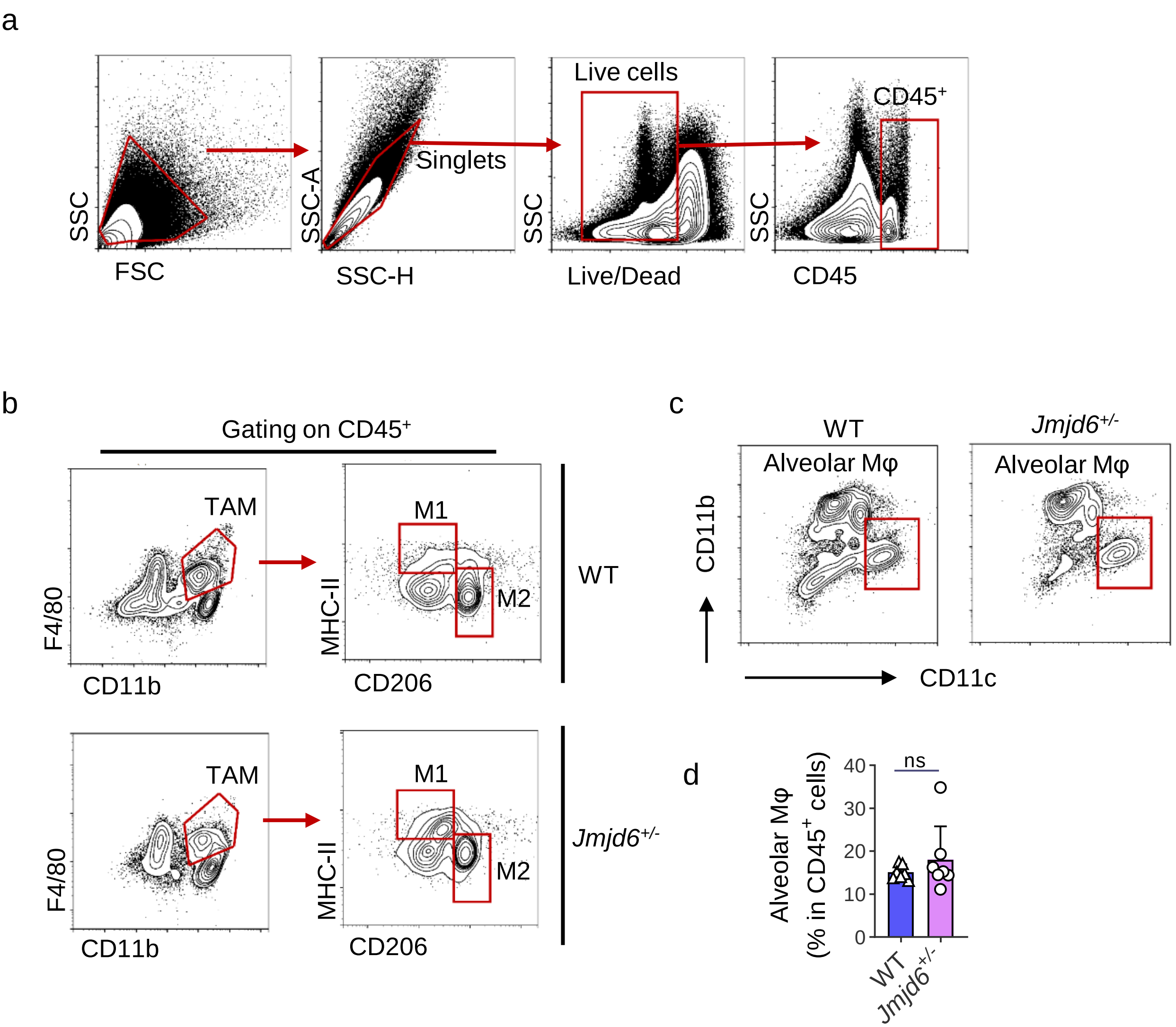

Supplementary Figure 6

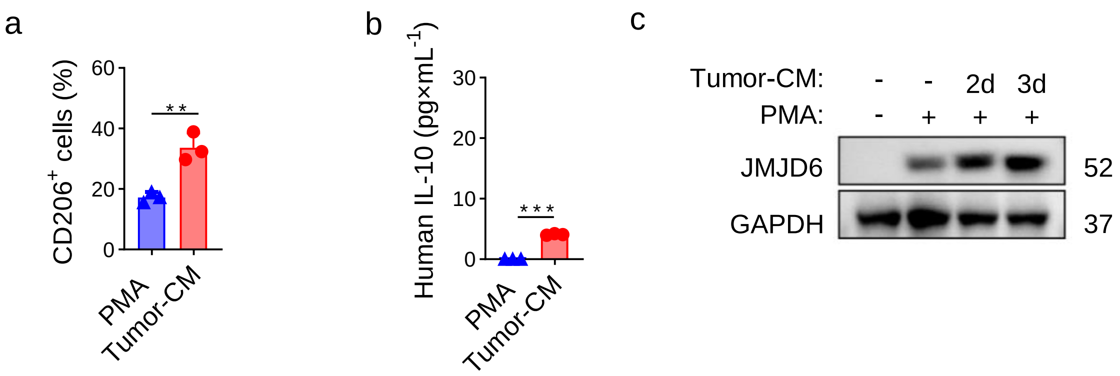

Supplementary Figure 7.

a

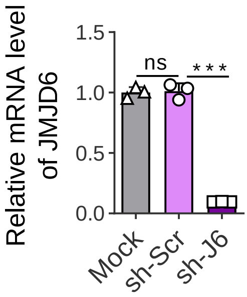

b

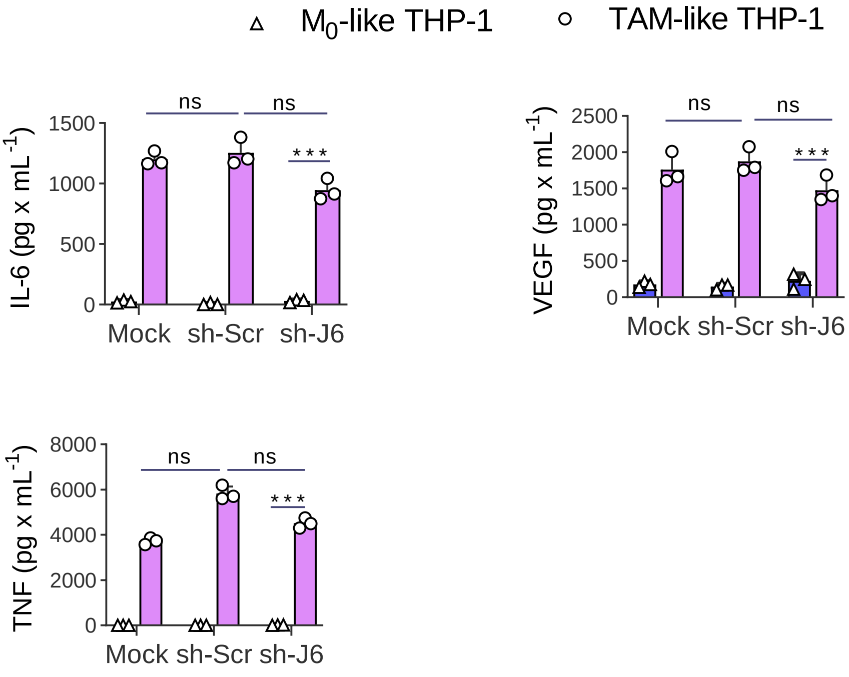

c

DEG Set	DEG Number	up-regulated	down-regulated
sh-J6 vs Mock	2765	1858	907
sh-Scr vs Mock	1798	1227	571
